# Psychological model of ART adherence behaviors in persons living with HIV/AIDS in Mexico: a structural equation analysis

**DOI:** 10.11606/S1518-8787.2017051006926

**Published:** 2017-08-25

**Authors:** José Luis Ybarra Sagarduy, Julio Alfonso Piña López, Mónica Teresa González Ramírez, Luis Enrique Fierros Dávila

**Affiliations:** I Unidad de Trabajo Social y Ciencias para el Desarrollo Humano. Universidad Autónoma de Tamaulipas. Ciudad Victoria, Tamaulipas, México; II Investigador independiente. Hermosillo, Sonora, México; III Facultad de Psicología. Universidad Autónoma de Nuevo León. Monterrey, NL, México; IV Departamento de Psicología y Ciencias de la Comunicación. Universidad de Sonora. Hermosillo, Sonora, México

**Keywords:** HIV Long-Term Survivors Acquired, psychology, Medication Adherence, Immunodeficiency Syndrome, drug therapy, Models, Psychological, Patient Medication Knowledge, Health Knowledge, Attitudes, Practice

## Abstract

**OBJECTIVE:**

The objective of this study has been to test the ability of variables of a psychological model to predict antiretroviral therapy medication adherence behavior.

**METHODS:**

We have conducted a cross-sectional study among 172 persons living with HIV/AIDS (PLWHA), who completed four self-administered assessments: 1) the Psychological Variables and Adherence Behaviors Questionnaire, 2) the Stress-Related Situation Scale to assess the variable of Personality, 3) The Zung Depression Scale, and 4) the Duke-UNC Functional Social Support Questionnaire. Structural equation modeling was used to construct a model to predict medication adherence behaviors.

**RESULTS:**

Out of all the participants, 141 (82%) have been considered 100% adherent to antiretroviral therapy. Structural equation modeling has confirmed the direct effect that personality (decision-making and tolerance of frustration) has on motives to behave, or act accordingly, which was in turn directly related to medication adherence behaviors. In addition, these behaviors have had a direct and significant effect on viral load, as well as an indirect effect on CD4 cell count. The final model demonstrates the congruence between theory and data (*x* ^2^/df. = 1.480, goodness of fit index = 0.97, adjusted goodness of fit index = 0.94, comparative fit index = 0.98, root mean square error of approximation = 0.05), accounting for 55.7% of the variance.

**CONCLUSIONS:**

The results of this study support our theoretical model as a conceptual framework for the prediction of medication adherence behaviors in persons living with HIV/AIDS. Implications for designing, implementing, and evaluating intervention programs based on the model are to be discussed.

## INTRODUCTION

In Mexico, since the beginning of the 2000s, significant effort has been made to provide persons living with HIV/AIDS (PLWHA) with antiretroviral medication in a free, timely way. Despite the evident advancements in this matter^1^, ironically, there has been little progress in the knowledge of how many PLWHA throughout the country are exhibiting medication adherence behaviors to antiretroviral therapy (ART)^2^. Except for one national study, which has included a representative sample of 2,054 PLWHA^3^, most of the studies have focused on samples of patients being treated in health care institutions, with an additional characteristic: studies are conducted without the support of a theoretical model that would predict how psychological or social variables influence the practice of medication adherence behaviors^4-6^.

**Figure 1:**
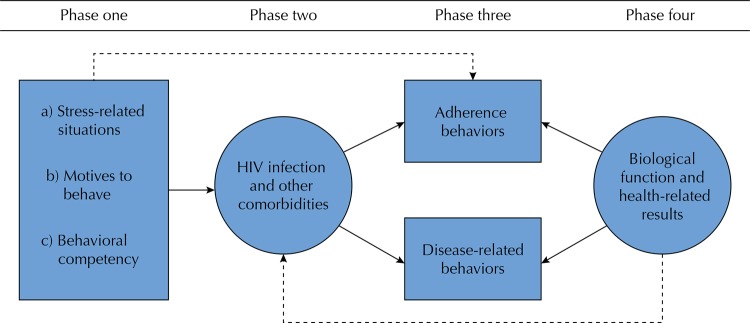
Psychological model for the research of adherence behaviors by Piña & Sánchez-Sosa9.

In the fields of psychology and health, such theoretical models are necessary because they help link basic and applied levels, in addition to having conceptual categories that allow researchers to predict whether or not a patient will practice ART adherence behaviors^7,8^. Based on this reasoning, years ago, a psychological model was proposed for the research of adherence behaviors in persons living with chronic diseases in general, and with HIV/AIDS in particular^9^. As shown in Figure 1, the model attempts to show the interaction of psychological and biological variables in four phases. Phase 1 has three psychological process variables: 1) personality or stress-related situations (including the behavior pattern of decision-making, tolerance of ambiguity, and tolerance of frustration), 2) the motives underlying the practice of adherence behaviors, and 3) behaviors related to past and current competencies. Phase 2 includes the HIV infection and other comorbidities. Phase 3 covers two types of behaviors: those related to therapeutics, and those related to the disease. Phase 4 includes conventional biological markers (CD4 lymphocytes and levels of viral load) and other health data.

Personality (in stress-related situations) is a process variable in which an individual displays patterns of behavior that are consistent over time. These patterns may give place to stress reactions when persons face situations in which: (a) the consequences of practicing a certain behavior are unpredictable and optional – *unpredictability*, (b) the stimuli signals to which the individual has to respond are ambiguous – *ambiguity*, and (c) the response to a situation with biologically harmful consequences, or the absence of them, do not depend on the individual’s behavior – *uncertainty*. These situations would make it possible to identify a functional or idiosyncratic profile of behavior, based on how each individual has interacted in the past with situations that are functionally similar in everyday life, or ones that are related to different diseases and their respective treatments^10,11^.

Motivation underlying behavior is a variable defined as an inclination or propensity to behave in a certain way in socially valuable circumstances. It is said that persons are motivated to engage in therapeutic adherence behaviors when they are willing to behave accordingly, after having understood the direct relationship between such behaviors and some positive outcome, such as an explicit expression of support, an economic reward, knowledge about a better understanding of their own health condition, etc^12^. Behavioral competencies is defined as the combination of knowledge, abilities, and skills acquired by an individual that allow him/her to respond in an effective way in situations where explicit instructions are given on what to do. Thus, it is said that a person is competent when he/she uses those resources to do what is expected, as would be the case in consuming medication as prescribed^13^.

Once individuals have been diagnosed with HIV, whether or not they effectively practice medication adherence behaviors, or, in this case, other behaviors stemming from their diagnosis (traditionally defined as anxiety, depression, rage, impulsiveness, etc.), will determine if they are more or less likely to maintain stable biological function or obtain optimal health results as a part of phase four. The latter will determine the clinical progression of the disease, whether positive or negative, and the eventual onset of other comorbidities, belonging to phase two. Of these comorbidities, in Mexico, we can point out cytomegalovirus, Kaposi’s sarcoma, *Pneumocystis jirovecii* pneumonia, tuberculosis, and cerebral toxoplasmosis. Finally, social support has been incorporated as a variable “exogenous” to the model, consistent with theoretical and conceptual considerations exposed in elsewhere^14,15^.

**Figure 2:**
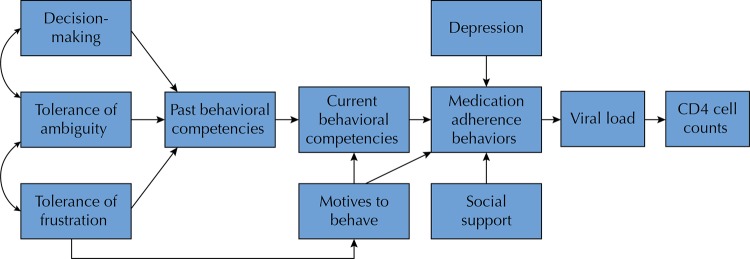
Hypothetical model of the relationships between variables and adherence behavior.

This study was conducted using the aforementioned psychological model. The objective was to identify which variables of the model, plus the variable of social support, would predict ART medication adherence behaviors in a group of PLWHA in the state of Tamaulipas, in northeast Mexico. According to the psychological model, Figure 2 presents a set of hypothetical relations between variables for the prediction of medication adherence behaviors. We expected a direct influence of the variable of personality on competence performance and motives, and their influence on medication adherence behaviors; moreover, we expected a direct influence of the variables of depression and social support on those behaviors; finally, we expected a direct influence of medication adherence behaviors on viral load and CD4 cell counts.

## METHODS

### Study Setting and Participants

This cross-sectional study was carried out in three Outpatient Clinics for the Prevention and Care of HIV/AIDS (CAPASITS) in Matamoros, Tampico, and Cd. Victoria, in the state of Tamaulipas, Mexico. CAPASITS in Tamaulipas provides specialized care for approximately 400 PLWHA, including patients receiving care in each center, and individuals recently diagnosed with HIV. Two hundred and nine patients were initially interviewed for eligibility purposes. The inclusion criteria to participate in this study were being: (a) aged 18 years or more, (b) HIV-positive or having received a confirmed AIDS diagnosis, and (c) under an ART treatment regimen, defined as the combination of at least two nucleoside and non-nucleoside inhibitors, plus one protease inhibitor. One hundred and seventy-two participants met the inclusion criteria, provided informed consent, and completed the assessment. In addition, information on biological measures (time since infection, viral load, and CD4 cell counts) was obtained from medical records. The study was approved by the Research and Ethics Committee Board of the *Universidad Autónoma de Tamaulipas*, as well as by the Research Committee Board of the Ministry of Public Health of Tamaulipas.

### Variables and Instruments

First, we used the Psychological Variables and Adherence Behaviors Questionnaire (VPAD-24), designed and validated in Mexico^16^. It consists of 24 questions grouped into four factors. For this study, factor one considered the measurement of medication adherence behaviors related to taking ART, and it was presented in a Likert-type format with five possible answers, ranging from one (“I did not take it on any day of the week”) to five (“I took it for seven days of the week”).

The second factor, motives*,* included four questions in a Likert-type format with four possible answers, ranging from one (“It was not a determining motive”) to four (“It was a determining motive”). One example of a question was: “Of the following motives, how much did each one influence you to take your medication every day of the week?” (e.g., “I took it because I have experienced its benefits in my health”); the lower the score, the less motivation to behave.

The performance factor of past competencies included five questions in a Likert-type format with five possible answers, ranging from one (never) to five (always). An example of a question was: “Before receiving a diagnosis of HIV seropositivity, when you had the flu, a sore throat, a stomach ache, etc., did you use to take medication as prescribed by your doctor?”

Finally, the performance factor of current competencies included four questions in a Likert-type format with four possible answers, ranging from one (very difficult) to four (not difficult at all). An example of a question was: “How difficult is it for you to follow your doctor’s instructions regarding taking your medication?”; the lower the score, the lower the current competency.

In addition, the variables of personality were measured against the Stress-Related Situations Scale (SVE-12), designed and validated in Mexico^17^. It consists of 12 questions grouped into three subscales: decision-making, tolerance of ambiguity, and tolerance of frustration. Each question was presented on a 10-point scale, in which 1 represented a situation considered as nonthreatening at all, and 10 as very threatening. An example of a question about *decision-making* was: “You are receiving treatment and your doctor gives you instructions that you must follow very carefully, but you decide to follow an alternative treatment (consuming homemade and holistic remedies), and you find that this seems…”. One example about *tolerance of ambiguity* was: “You have been diagnosed with a disease for which you do not know if there will be a cure in the short or long-term, but you must follow a treatment plan, and you find that this seems…”. One example about *tolerance of frustration* was: “You are receiving treatment and do everything you are told to do, but you do not receive positive results from healthcare personnel, and you find that this seems…”. The lower the score was, the lower the evaluation of stress in each situation proved to be.

Furthermore, we used the Zung Depression Scale, previously adapted and validated in Mexico^18^. It includes 20 questions about physical and psychological symptomatology in the last 30 days (e.g., I notice that I am losing weight; I have trouble sleeping; I feel bored, discouraged, and sad; among others). Each question was designed in a Likert-type format with four possible answers, ranging from one (very few times) to four (most of the time). A lower score meant lower levels of depressive symptomatology.

Finally, we used the Duke-UNC Functional Social Support Questionnaire, previously adapted and validated in Mexico^19^. It includes 11 questions that measure the perceived support of significant persons in the patient’s social environment (e.g., my family and friends come to visit me). The questions are in a Likert-type format of four points, ranging from one (less than I wish or expect) to four (more than I wish or expect). A lower score meant less perceived social support.

From medical records, we obtained information about the ART regimen and the main biological variables at work, which included the length of the infection in months, as well as the levels of viral load and CD4 cell counts. Length of infection was defined as the time elapsed in months since the diagnosis of HIV seropositivity. Viral load was defined as the amount of copies of virus/ml, considering the register < 400 copies/ml as an indicator of virus suppression. The CD4 cell counts were defined as the amount of cells/mm^3^, considering an optimal indicator those patients who registered > 201 cells/mm^3^.

### Data Analysis

A first analysis was carried out to obtain descriptive statistics – means, standard deviations (SD), and minimum and maximum values of the answers – from each of the independent variables. Immediately afterwards, the bivariate analysis was carried out with Pearson’s correlation coefficient, which included the medication adherence behaviors and the variables of the model, as well as the variable of social support. Finally, using structural equation modeling, several trajectory analyses were conducted, with which we can specify the direct and indirect relationship among variables^20^. Following recommendations on the matter, different indicators of goodness of fit were used: Chi-square (*x* ^2^), the relationship between Chi-square and degrees of freedom (*x* ^2^/df, whose value must be ≤ 3), the goodness of fit index (GFI), the adjusted goodness of fit index (AGFI), the comparative fit index (CFI), and the root mean square error of approximation (RMSEA). Non-significant values of chi-square, above 0.90 in GFI, AGFI, CFI, as well as those under 0.80 in RMSEA, combined, reflected the accuracy of the data in the model^21,22^.

## RESULTS

### Measures and Values

As shown in Table 1, means and SD are specified for the different assessment instruments, as well as the maximum and minimum possible scores, the scores obtained, and the values of Cronbach’s Alpha (α) for each variable, as they were grouped in their respective subscales.

**Table 1:** Descriptive statistics of the study variables and Cronbach’s alpha of 172 persons living with HIV/AIDS in Tamaulipas, Mexico.

Measure	Mean	SD	Range (Possible)	Range (Current)	Cronbach’s alpha (α)
Decision-making	13.0	10.2	3–30	3–30	0.83
Tolerance of ambiguity	16.9	9.9	3–30	3–30	0.80
Tolerance of frustration	23.9	15.3	6–60	6–60	0.82
Motives to behave	11.4	5.7	4–16	4–16	0.92
Past competencies	16.2	7.2	5–25	5–25	0.88
Current competencies	17.0	3.7	4–20	4–20	0.75
Depression	40.0	8.8	20–80	20–69	0.76
Social support	47.9	9.5	11–55	11–55	0.87

### Sociodemographic, Clinical, and Behavioral Characteristics of the Participants

The average age of the 172 PLWHA of this sample (Table 2) was 37.9 years (95%CI 36.4–39.5). Most of the participants were men (n = 106), unmarried (n = 96), and had less than nine years of formal education (n = 126). The average time they had been living with HIV was 51.1 months (95%CI 44.9–58.4). In addition, 145 of them (84.8%) presented undetectable levels of viral load or ≤ 400 copies/ml, while 151 (88.3%) registered CD4 cell counts equal or superior to 201 cells/mm^3^. On the other hand, 141 (82%) participants stated they were 100% adherent to ART, that is, they answered that they had taken the medications as they had been prescribed during the seven days prior to the study.

**Table 2:** Sociodemographic, clinical, and behavioral characteristics of 172 persons living with HIV/AIDS in Tamaulipas, Mexico.

Variable	n*	%*
Age (years)*	37.9 (20–72)	36.4–39.5
Gender		
Male	106	61.6
Female	66	38.4
Marital status		
Single	96	55.8
Married or cohabitating	71	41.3
Missing data	5	2.9
Education status (years)		
Illiterate	3	1.7
Less than 9 years	126	73.2
More than 9 years	42	24.4
Missing data	1	0.5
Occupation		
Employed	59	34.3
Unemployed	113	65.7
Time since infection (months)*	51.5 (6–40)	44.9–58.4
Viral load (copies/ml)		
Undetectable	145	84.8
Between 401–30,000	5	2.9
More than 30,000	20	11.7
Missing data	1	0.6
CD4 cell count (cell/mm^3^)		
Less than 200	20	11.7
Between 201–400	73	42.7
More than 400	78	45.6
Medication adherence behaviors		
100% adherents	141	82.0
Non-adherents	31	18.0

* For the variables of age and time since infection, the values are presented in mean (range) and 95%CI.

### Bivariate Analyses

Table 3 shows the correlations between the study variables. Only one correlation between the medication adherence behaviors and the variable of motives to behave was found (r *=* 0.751). In addition, for the variable of personality, we can point out the correlation between decision-making and tolerance of ambiguity (r = 0.599), tolerance of frustration (r = 0.635), and social support (r = 0.184), as well as between tolerance of ambiguity and tolerance of frustration (r = 0.710) and depression (r = 0.162), and, in a negative sense, between tolerance of frustration and the variable of motives to behave (r = -0.204). Neither the variables of past competencies nor the variables of current competencies showed a correlation with any of the stress-related situations or the variable of motivation to behave; furthermore, current competencies negatively correlated with depression (r = -0.640), and positively with social support (r = 0.462). Finally, depression and social support had negative correlation (r *=* -0.396).

**Table 3:** Correlation matrix of psychological variables, social support, and medication adherence behaviors of 172 persons living with HIV/AIDS in Tamaulipas, Mexico.

Variable	2	3	4	5	6	7	8	9
1. Adherence behaviors	0.170	-0.042	-0.113	0.751^b^	0.045	0.135	0.019	0.023
2. Decision-making		0.599^b^	0.635^b^	0.008	-0.002	-0.099	0.138	0.184^a^
3. Tolerance of ambiguity			0.710^b^	-0.146	0.061	-0.097	0.162^a^	0.128
4. Tolerance of frustration				-0.204^b^	-0.014	-0.109	0.133	0.078
5. Motives					-0.096	0.009	-0.051	0.011
6. Past competencies						-0.017	0.058	-0.007
7. Current competencies							-0.640^b^	0.462^b^
8. Depression								-0.396^b^
9. Social support								

^a^ p < 0.05

^b^ p < 0.01

### Structural Equation Modeling

Considering the hypothetical relationships described in Figure 2, in the first model we included the variables of the theoretical model and social support. The significant parameters in this model were the direct effect of decision-making (β = 0.233; p = 0.018) and tolerance of frustration on the variable of motives to behave (β = -0.285; p = 0.009). In addition, high motivation was directly related to medication adherence behaviors (β = 0.745; p = 0.001), which were also related to lower levels of viral load (β = -0.319; p = 0.001), which, in turn, showed a direct effect on CD4 cell counts (β = -0.302; p = 0.018). However, the results of the remaining parameters were not significant, and the indicators of goodness of fit were not optimal (GFI = 0.84; AGFI = 0.75; CFI = 0.72; RMSEA = 0.13). For this reason, we proceeded to look for a model with better fit, setting the different parameters that were not significant in the prior modeling to zero.

**Figure 3:**
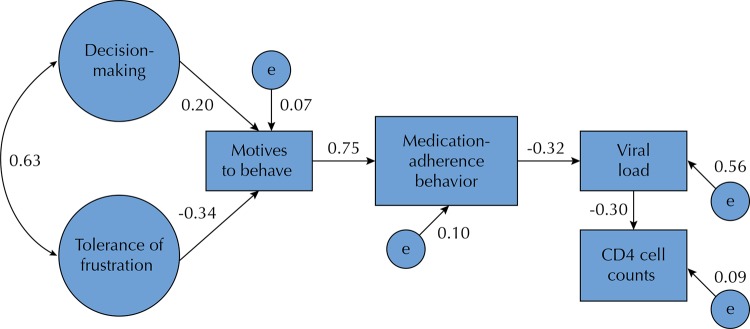
Path analyses of medication adherence behavior among 172 persons living with HIV/AIDS in Tamaulipas, Mexico.

As shown in Figure 3, the second analysis resulted in a model with better fit. We found that two stress-related situations, decision-making (β = 0.202; p = 0.033) and tolerance of frustration (β = -0.344; p = 0.001), had a direct effect on motives to behave, explaining the 7.2% variance between them. The variable of motives to behave had a direct and significant effect on medication adherence behaviors (β = 0.746; p = 0.001), mediating the influence of the two stress-related situations, decision-making (β = 0.150; p < 0.05) and tolerance of frustration (β = -0.257; p < 0.05), which together, explain a 55.7% variance. In addition, medication adherence behaviors, in turn, showed a direct and significant effect on lower levels of viral load (β = -0.317; p = 0.001), which directly affected CD4 cell counts (β = -0.302; p = 0.001), explaining the 9.1% variance between them. For the estimation of the model, we used the method of maximum likelihood. The data were adjusted for the multivariate normality and we used scores corresponding to the totals obtained in the different scales and subscales. The statistics of goodness of fit for the final model were optimal in every case: x^2^/df. = 1.480; GFI = 0.97; AGFI = 0.94; CFI = 0.98; RMSEA = 0.05.

Goodness of fit: (x^2^/df. = 1.480; GFI = 0.97, AGFI = 0.94, CFI = 0.98; RMSEA = 0.05).

Solid lines indicate significant relationships (p < 0.05).

## DISCUSSION

The goal of this study was to test the influence of variables of a psychological model and the role of social support on medication adherence behaviors related to ART in a sample of 172 PLWHA in the state of Tamaulipas, Mexico. One hundred and forty participants reported being 100% adherent, that is, they had consumed all medications as prescribed during the week prior to the study. Structural equation modeling revealed the direct influence of two stress-related situations on the motives underlying the practice of medication adherence behavior. The results partially coincide with those reported among PLWHA in the state of Sonora, in northwest Mexico^23^, even though they are slightly different, since in this sample there is no evidence of direct influence of the variable of motives to behave on behavioral competencies, or the latter on medication adherence behaviors.

Personality (stress-related situations) is defined as a consistent way of behaving in circumstances in which the consequences of a response cannot be predicted (decision-making), or in which the consequences of a certain behavior do not depend on the actions of the PLWHA (tolerance of frustration). In this situation, we are addressing the most deeply rooted variable in a person’s life, which is crucial for the prediction of medication adherence behaviors. Decision-making related to therapy can sometimes compete with the practice of adherence behavior^24,25^, because when PLWHA face situations that involve two or more simultaneous options (taking the medication or not, or more specifically, following orders to further take them or not), such conditions can provoke stressful reactions with variable length. In this sample, we found that, in general, participants showed low levels of stress related to decision-making, judging by the mean response values for this variable (13.0 points on a scale ranging from 3 to 30 points). Along the same lines, there are also those who face situations in which a specific activity is carried out (taking the medications as prescribed by healthcare personnel), but the result (in the form of reinforcing stimuli) is less than expected, is delayed, is not granted, is taken away without reason, etc. The result is thus the manifestation of behavioral patterns that exhibit low tolerance of frustration^26,27^. Both of these stress-related situations also had a direct influence on the motives underlying medication adherence behaviors.

It is important to remember that the variable of motives to behave is related to the most current, cumulative^11^, and changing background of each individual patient. In this item, the questions concerning the reasons for practicing medication adherence behaviors were related to intrinsic stimulus (having knowledge about an optimal health condition and experiencing it), and extrinsic stimulus (receiving verbal manifestations of recognition or positive results, such as rewards from healthcare personnel or other significant persons in the patient’s social environment).

As exposed in Table 1, the participants have an optimal level of competences, as they know about their health condition, but at the same time they know what do with that information, to identify which positive results are associated with the practice of medication adherence behaviors. Knowing the causes or the characteristics of the disease and treatment seems to have an influence on how certain actions are performed, which, in this case, would include taking the ART according to instructions given by the healthcare team. Nevertheless, it is worth mentioning that past and current competencies did not have a significant impact on the practice of medication adherence behaviors, particularly if we consider two facts in relation to other studies, based in our theoretical model. These facts determined a solid relationship between motives to behave and behavioral competencies, especially when we worked with samples of individuals who were 100% adherent or who had high percentages of adherence^28,29^, such as is the case of our study; in this study, 141 (82%) of the participants reported being 100% adherent to ART.

On the other hand, despite having found low levels of depression and high scores of perceived social support, contrary to what has been reported in previous studies^30-33^, neither variable played a significant role in any of the structural models analyzed. This is a particularly intriguing result, as the first score was 16.2 and 17.0 for past and current competencies (of a possible maximum of 25 and 20, respectively), which would reflect an optimal competence performance in both moments. Meanwhile, the second score was 40 (of a possible range of 20 to 80), which we could say is equivalent to a “moderate” level of depression. Regarding social support, the mean score was high – it reached 47.5 (of a possible range of 11 to 55) –, meaning that the participants in this sample perceived exceptional social support rom information and trust. One possible explanation would perhaps be the fact that the study was carried out with a sample of PLWHA who were mostly 100% adherent, that is, with persons who have proved to be competent and have significant sources of social support.

In this study, we worked with a sample of PLWHA that had been living with the disease for an average of 51.5 months, in this way it is possible to argue that, as more time with the infection passes, and if the individual is not transitioning towards AIDS, the psychological or social variables will have a greater or smaller impact, just as it has been reported in several studies which included PLWHA from different regions of Mexico^34-36^. This means that, if PLWHA have, at this time, already undergone treatment programs, and at the same time have been exposed to intervention programs carried out at health institutions, it is reasonable to believe that they are more likely to be psychologically and socially accustomed to the disease and to ART, which is seen as a necessary requirement for subsequent medication adherence behaviors^37,38^.

Finally, an issue that cannot be overlooked and that reveals the influence of the two stress-related situations on stress and motivation is that, (a) knowing how to face such situations, and (b) doing things based on a specific motivation are both critical elements in the prediction of adherence behaviors. In addition, as described in the results section, these elements also have subsequent influence on lower levels of viral load and high CD4 cell counts. This finding is consistent with information reported in studies carried out by other authors^39,40^. In summary, it is understandable that, in this sample, optimal adherence resulted in low levels of viral load and high CD4 cell counts. We must keep in mind that 84.8% of the PLWHA presented low levels of viral load (undetectable or < 400 copies/ml), and 88.3% obtained high CD4 cell counts (> 201 cells/mm^3^).

Even with the results obtained in this study, it is important to recognize some limitations. The first limitation includes the size of the sample, which consisted of approximately 40% of PLWHA exposed to treatment regimens with antiretroviral medication in the five CAPASITS centers in the state of Tamaulipas, in northeast Mexico. The second one, because this is a sample in which most of the PLWHA reported being 100% adherent to antiretroviral therapy (n = 141; 82%), we have to consider a likely overestimation in the self-reporting of the practice of adherence behaviors. Finally, the lack of influence of the variables of behavior competencies, depression, and social support is also worth noting. In previous studies with high or 100% adherence samples, one of the key variables has been the variable of behavioral competence. However, as mentioned, at least in this sample, no influence was found, since two of the variables related to personality (decision-making and tolerance of frustration) played a role of first order^28,29^.

For such reasons, and according to previous discussion on this topic, further studies need to be carried out using a broader sample of PLWHA from different regions of the country. Our current theoretical model will undergo strict empirical tests in future research, comparing PLWHA who (a) are adherent or have adherence problems, (b) have demonstrated the pertinent behavioral competencies or not, and (c) show particular symptoms of depression or have established social support networks. This last aspect is especially important because the results would allow us to design, implement, and evaluate specific intervention programs based on the concrete needs of patients in the respective institutions where they are routinely treated.

## CONCLUSIONS

This transversal study revealed the influence of two stress-related situations (decision-making and tolerance of frustration) on the underlying motives of ART medication adherence behaviors in a sample of 172 PLWHA in the state of Tamaulipas, Mexico. These situations also had an influence on decreased levels of viral load and high CD4 cell counts. Variance, in combination with the variables of the theoretical model on which the study was based, reached 55.7%. Something worth mentioning is the lack of influence of the variables of depression, social support, and motives to behave on behavioral competencies. In this context, the results contribute to empirically validate our theoretical model and to explore theoretical intervention options in order to increase the practice of medication adherence behaviors in patients.

Therefore, this would also mean trying to establish guidelines that would guarantee the relationship between the conceptual categories of the model and the procedures and techniques from a behavioral perspective.
